# Experimental Study on Fabrication of CVD Diamond Micro Milling Tool by Picosecond Pulsed Laser

**DOI:** 10.3390/mi12091058

**Published:** 2021-08-31

**Authors:** Yi Xia, Ning He, Liang Li, Guolong Zhao

**Affiliations:** College of Mechanical and Electrical Engineering, Nanjing University of Aeronautics and Astronautics, Nanjing 210016, China; xiayi@nuaa.edu.cn (Y.X.); drnhe@nuaa.edu.cn (N.H.); zhaogl@nuaa.edu.cn (G.Z.)

**Keywords:** micromachining, CVD diamond, micro milling tool, picosecond pulsed laser, laser cutting

## Abstract

Because of the many advantages of high-precision micromachining, picosecond pulsed lasers (PSPLs) can be used to process chemical-vapor-deposited diamonds (CVD-D). With the appropriate PSPL manufacturing technique, sharp and smooth edges of CVD-D micro tools can be generated. In this study, a PSPL is used to cut CVD-D. To optimize PSPL cutting, the effects of its parameters including fluence, pulse pitch, and wavelength on the cutting results were investigated. The results showed that the wavelength had the greatest impact on the sharpness of CVD-D. With PSPL cutting, sharp cutting edges, and smooth fabricated surfaces of the CVD-D, micro tools were achieved. Finally, the fabrication of CVD-D micro milling tools and micro milling experiments were also demonstrated.

## 1. Introduction

The hardness, molar density, thermal conductivity, bulk elastic modulus, and acoustic velocity of diamonds are higher than other natural materials, and its compressibility is lower than other materials. The diamond also has a very low thermal expansion coefficient of 0.8 × 10^−6^ at 293 K [1] and excellent optical and electrical properties. There are two kinds of diamond materials: natural and synthesized. However, because of the scarcity of natural diamonds, synthetic diamonds have become particularly important. Synthetic diamonds can also be classified into many types. The chemical-vapor-deposited diamond (CVD-D), poly-crystalline diamond (PCD), and single crystal diamond (SCD) are the most widely used types in industry. Compared to PCD, CVD-D is harder and exhibits a lower friction coefficient, higher abrasion resistance, higher thermal conductivity, and better chemical and thermal stability. Compared to SCD, isotropic CVD-D cannot cleave along the crystal plane, which severely restricts the use of SCD.

Because of its excellent properties [2,3], CVD-D is a promising material for numerous mechanical, thermal, optical, electrical, and electronic applications. It has been widely used in modern industrial products such as cutting tools, semiconductors, optical windows [4,5,6], heat spreaders, X-ray lithography, and active electronic device elements [1]. Micro manufacturing is an important part of ultra-precision machining. In micro fabrication, with the micro milling tool, a smooth surface can be obtained with good work quality and high processing efficiency, and CVD-D is a suitable material for producing micro milling tools [7,8].

Diamonds can be fabricated by traditional grinding and nontraditional processing such as electrical discharge machining (EDM) [9,10], electrochemical machining (ECM), electrochemical discharge machining (ECDM), and laser machining [11]. Because of the weak rigidity, extreme hardness, and wear resistance of CVD-D material, grinding it may cause severe cracks and even breakage on the cutting edge. EDM and ECM are not suitable for processing CVD-D because of the poor conductivity. Long pulsed lasers can produce excessive heat effects on CVD-D, so sharp cutting edges and smooth fabricated surfaces are difficult to obtain. The ultrashort pulsed laser (pulse duration < 15 ps) process has advantages such as force-free and wear-free material removal, a lower heat effect on processed material, etc., which makes it an ideal choice for processing CVD-D with high efficiency and accuracy.

Ultrashort pulsed laser fabrication of diamonds has been investigated over a wide range of pulse durations and wavelengths. Weikert and Dausinger [12] discovered that using ultrashort pulsed lasers with pulse durations of 120 fs to 2 ps is a viable technique to cut SCD, PCD composites, and CVD-D. For cutting SCD, the laser pulse duration should remain below 1 ps, but the PCD composite and CVD-D can be fabricated by a laser with a pulse duration of 2 ps. Joswig et al. [13] used a laser with a pulse duration of 150 to 250 fs and a wavelength of 800 nm to generate or modify tool tips on CVD-D and SCD for turning and milling applications. It was concluded that when using femtosecond pulse durations, the quality of the surface and form of the structures were quite good, but this was not applicable to direct diamond cutting for optical applications. Dold et al. [14] used a laser with a pulse duration of 10 ps and a wavelength of 1064 nm for ablation processing on fine and coarse grain PCD cutting tools. Cutting edge radii for coarse grain laser-treated PCD tools were in the range of 4 < r_edge_ < 8 μm, and no significant material damage on the cutting edge was found. When using laser rather than grinding processing, the machining times are slightly shorter in cutting edge generation. Eberle et al. [15] demonstrated that when processing PCD composites with a laser with a pulse duration of 10 ps, no residual graphitic carbon layer is present, contrary to nanosecond and microsecond pulses. Yoshinori et al. [16] studied the ablation effect on the binder-less polycrystalline diamond (BLPCD) by femtosecond pulsed lasers with wavelengths of 1045 and 522 nm. It turned out that the femtosecond pulse laser can be utilized for successful machining of BLPCD, resulting in 22 nm average surface roughness with almost no graphitization surface layer.

In this paper, considering the excellent processing performance of the picosecond pulsed laser (PSPL) and the potential advantages of lasers with different wavelengths in CVD-D machining, we used infrared and ultraviolet PSPL to process CVD-D micro tools and investigated the influence of PSPL processing parameters on the achievable cutting edge radius and fabricated surface. CVD-D micro milling tools with diameters of 200 and 600 μm were fabricated by PSPL using the optimized laser cutting parameters.

## 2. Design of Experiments

An infrared picosecond pulsed laser and an ultraviolet picosecond pulsed laser were used for the experimental work, and the output wavelengths were centered at λ = 1064 nm and λ = 355 nm, respectively. Table 1 lists the main parameters of the lasers. The laser micromachining center was used for the test. The picosecond pulsed laser system was integrated into the laser machining center, and the laser beam was collimated by a beam expander and conducted to the scanner (SCANLAB intelliSCAN 14) through several bending mirrors. The galvanometer motions in the scanner reached the marking speed of 2 m/s on the workpiece surface. The repetition rate of the lasers was set as 20 KHz.

**Figure 1:**
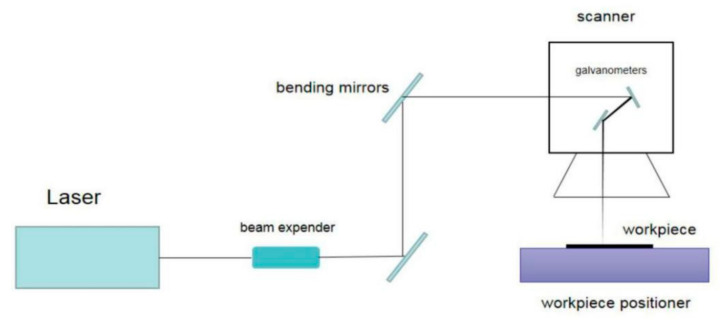
Diagrammatic sketch of the laser cutting method.

**Figure 2:**
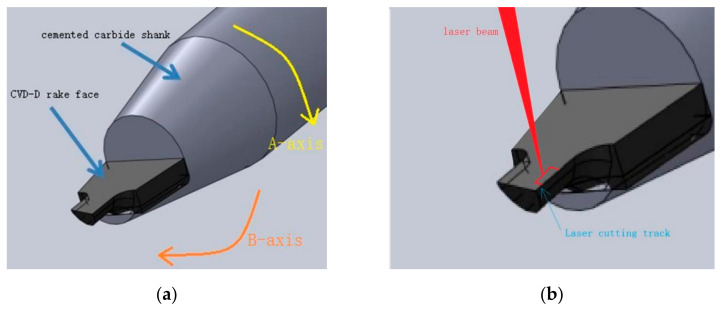
The laser cutting method: (**a**) CVD-D micro tool blank; (**b**) Schematic diagram of laser cutting.

CVD-D micro tools with lapped surfaces were used in the experiment. As shown in Figure 1, the CVD-D micro tools were placed on the objective stage of the machining center, and the F-theta lens of the scanner focused the laser beam onto the rake face of the CVD-D tool blank from the vertical direction. Figure 2 shows the laser cutting method of CVD-D, the laser scanned along the cutting track.

To optimize the PSPL cutting process, a single factor test was utilized to plan this experiment. We chose the fluence, pulse pitch, and wavelength of the PSPL as the process parameters influencing the cutting performance. The Pulse pitch is defined as the distance between two adjacent laser pulses, which can also be defined with Equation (1).

Pulse pitch = *v*/*f* (1)

where *v* is scan speed of laser (mm/s), and *f* is pulse repetition rate (s^−1^).

A design of experiment was implemented so that the parameters to identify the ones affecting the cutting performance most significantly were accounted for. In order to compare the laser cutting results effectively, the focusing spot diameters of the two lasers were adjusted to be the same (16 μm). Table 2 lists the cutting parameters and corresponding levels. Fluences from 12.4 to 24.9 J/cm^2^, and a pulse pitch from 0.5 to 5 nm, were chosen for the PSPL cutting of CVD-D micro tools.

In micro machining, because of the size effect, the cutting performance of the CVD-D micro tool is greatly affected by the edge sharpness. Thus, the cutting edge radius was a major criterion. The quality of the fabricated CVD-D surface also affects its performance. Thus, surface quality was another criterion.

The cutting edge radius (r_n_) of processed CVD-D was measured with a confocal microscope with a resolution of 1 nm. The roughness of the CVD-D surface was measured with a three-dimensional laser scanning confocal microscope (Phase Shift Micro XAM-3D). The morphology of the tools and the fabricated surfaces were observed using scanning electron microscopes (SEM, Hitachi S-3800 and S-4800).

## 3. Results and Discussion

A smaller cutting edge radius of the micro tool results in better micromachining performance because of the size effect. Therefore, our goal of the research was to obtain a sharper cutting edge. The results for the cutting edge radius are shown in Table 3. We found that the cutting edge radius of the second set of experiments had the minimum value. To optimize the laser cutting parameters independently, X_1_, X_2_, and X_3_ were taken as the average values of the results corresponding to the level 1, 2, and 3 of each parameter. The meaning of “Max-min” is the range of the values of Xn. The smaller the average value in each level, the better the cutting edge quality characteristic. Therefore, the laser process parameters with the smallest cutting edge radius were as follows: fluence (12.4 J/cm^2^), pulse pitch (2.5 nm), and wavelength (1064 nm).

**Figure 3:**
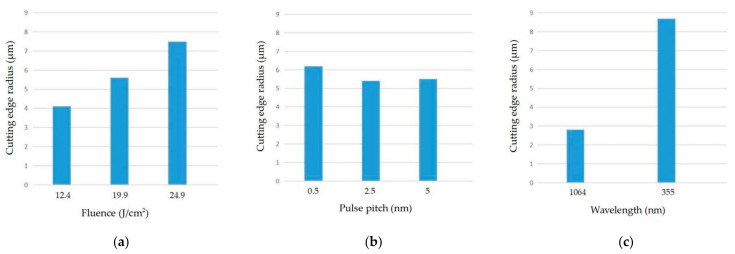
The experimental results of the cutting parameters on the edge radius: (**a**) fluence; (**b**) pulse pitch; (**c**) wavelength.

The effect trends of the laser cutting parameters on the cutting results are shown in Figure 3. For less pulse fluence, resulting in a sharper cutting edge, refer to Figure 3a. For the moderate pulse pitch, the interaction between the laser and diamond has more often than not a shorter pulse pitch. In addition, increasing the pulse pitch leads to a decrease in laser cutting fluence, and this can lead to a decrease in the edge integrity of the diamond and enlarge the cutting edge radius; see Figure 3b. The results represented in Figure 3c show that the laser with the larger wavelength has a better cutting result.

Variance analysis was used to quantify the contribution of the PSPL cutting parameters in the results. It was used to identify and quantify the contributions of the different parameters in the results. The influence of the parameters on the cutting edge quality was obtained. Table 4 illustrates that for CVD-D, the wavelength (84.9%) and pulse pitch (0.9%) of the PSPL had the greatest and least effect on the cutting edge radius characteristic, respectively.

**Figure 4:**
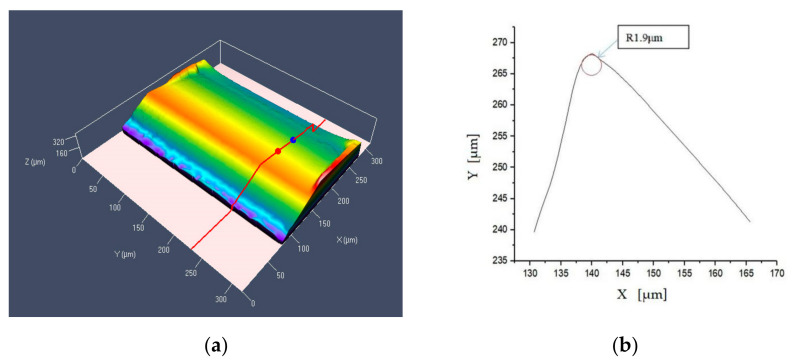
The surface topography and profile of CVD-D, (**a**) 3D profile, (**b**) 2D profile.

**Figure 5:**
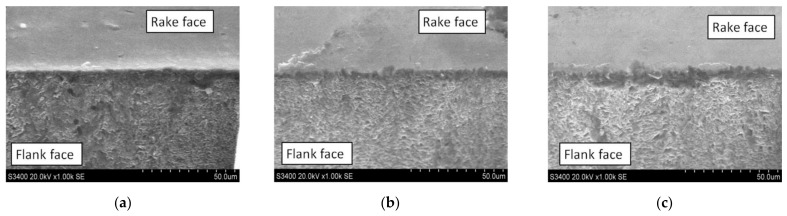
CVD-D micro tools after infrared PSPL cutting at (**a**) 12.4 J/cm^2^; (**b**) 19.9 J/cm^2^; and (**c**) 24.9 J/cm^2^.

Figure 4 shows the typical surface topography and profile of the measured cutting edge from the first set of parameters. The ×2000 SEM magnification images of the cutting edges of CVD-D are shown in Figure 5.

As shown in Figure 5, the cutting edge fabricated with the fluence of 12.4 J/cm^2^ was much sharper and more regular than the cutting edge fabricated with the fluence of 19.9 and 24.9 J/cm^2^, and fewer cracks were found on the cutting edge. When the fluence was too large (24.9 J/cm^2^), obvious cracks can be found; the size of the cracks is approximately 5 μm.

The PSPL processing of CVD-D is divided into several steps. The first is the Coulomb explosion caused by cumulative ionization and multi-photon ionization. When the laser fluence exceeds the threshold of CVD-D, tiny etching pits appear on the surface of the diamond. Other steps are explosive boiling, the critical point phase, and atomisation which had been demonstrated by Eberle [17].

The Coulomb explosion has great impact on the formation of and damage to the cutting edge. Lower fluence causes less damage to the edge of diamond. When the fluence is too large and absorbed by the material, the Coulomb explosion is more intense, resulting in slight breakage of the diamond edge, which leads to the decrease in cutting edge quality. With the increase in fluence, the thermal damage to the diamond ablation area increases correspondingly, and the laser-processed cutting edge radius becomes larger.

**Figure 6:**
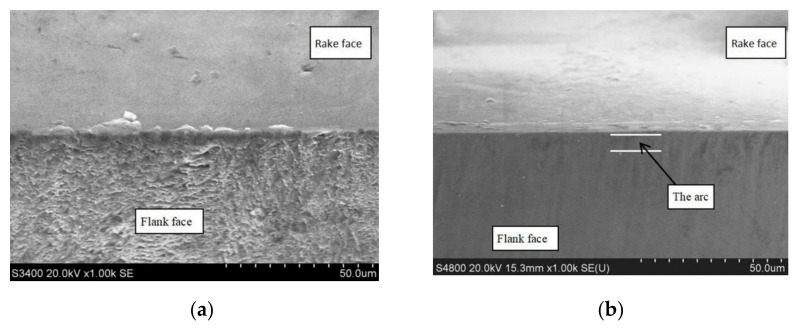
CVD-D micro tools processed by infrared and ultraviolet PSPL, (**a**) infrared PSPL cutting, (**b**) ultraviolet PSPL cutting.

**Figure 7:**
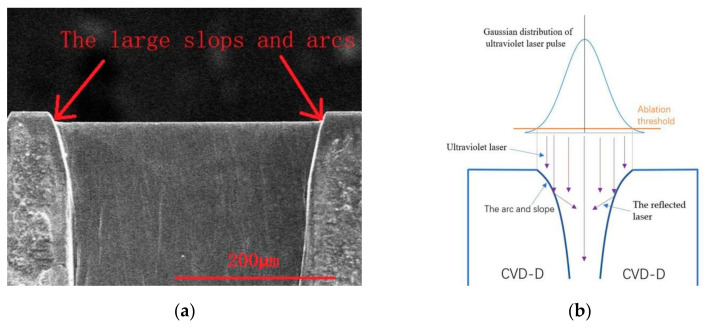
(**a**) SEM image of the slops and arcs produced by ultraviolet PSPL; (**b**) Schematic illustration of the formation of slopes and arcs.

The PSPL wavelength also has a severe influence on the cutting edge quality. As shown in Figure 6 and Figure 7a, CVD-D had been cut by lasers at two different wavelengths with the same fluence of 12.4 J/cm^2^ and pulse pitch of 2.5 nm; after ultraviolet PSPL cutting, an arc with a large radius was formed between the rake face and the flank face of the CVD micro tool. Because of the high absorptivity of CVD-D of the ultraviolet laser [18,19], the laser ablated the diamond surface severely, and the laser beams with the Gaussian distribution produced large slopes along the cutting edge. The slopes then reflected the subsequent incident lasering and concentrated the laser energy at the bottom of the slopes, which caused the large cutting edge radius. In addition, the formation of slopes and arcs is displayed in Figure 7b.

**Figure 8:**
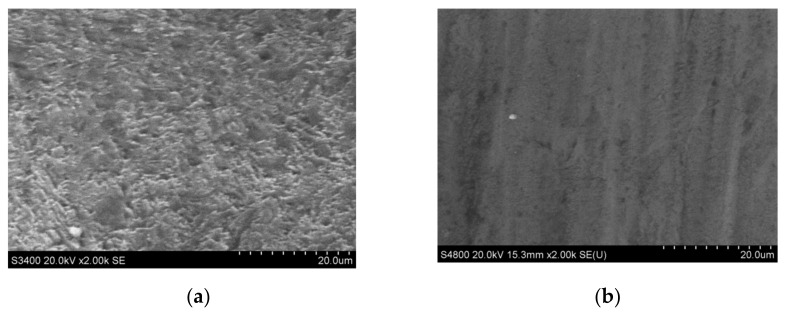
CVD-D micro tools after PSPL cutting with different wavelengths, (**a**) 1064 nm, (**b**) 355 nm.

The surface quality of CVD-D micro tools was analyzed after PSPL cutting. The surface geometries obtained after PSPL cutting with the same fluence (12.4 J/cm^2^) and pulse pitch (2.5 nm) but different wavelengths are shown in Figure 8. After ultraviolet PSPL and infrared PSPL cutting, the Ra value of the fabricated surface was 149 and 311 nm, respectively, and there were fewer defects on the ultraviolet laser-fabricated surface. This was the result of the different thermal and photochemical effects of the infrared and ultraviolet laser on the diamond. During the infrared PSPL processing, the interaction between laser and material was mainly a photothermal effect. When the infrared laser beam irradiated the surface of the diamond micro tools, part of the photon energy was absorbed by the electrons on the surface, which increased the electron kinetic energy. Through thermal relaxation and heat transfer, the temperature of the irradiated area of the diamond rose, and the material vaporized. Ultraviolet PSPL pulses, however, have high single photon energy (3.5 eV), and attain high peak intensities resulting in high photon density. According to Eberle [17], the photons to bridge bandgap of diamonds are 2 and 5 with ultraviolet and infrared PSPL, respectively. Therefore, ultraviolet PSPL can bridge the bandgap and lead to multiphoton absorption with a higher probability since fewer photons are required, and photon energy seldom converted into heat energy, so less thermal damage is caused to the diamond grains. Concurrently, the surface roughness of the diamond fabricated by the ultraviolet laser is lower.

The laser-induced graphitized layer occurred on the clearance face of CVD-D under its ablation by the infrared PSPL [20,21], as shown in Figure 8a. It is believed that the initial surface graphitization of the diamond is initiated through the absorption of laser radiation by the present structural defects and nondiamond inclusions and leads to the formation of a comparatively homogeneous layer of nanocrystalline graphite on the surface. The graphitic carbon also arises from the recasting of the vaporized diamond in the form of amorphous carbon when the CVD-D is processed under tangential irradiation of the laser. After the graphitized layer is formed, it moves deep into the diamond with the action of each subsequent laser pulse because of sublimation of the substance from the surface; this is accompanied by a reconstruction of the carbon crystal structure at the interface between the graphite-like phase and diamond phase. The thickness of the graphitized layer is determined by the depth of the heat-affected zone, radiation absorption depth of the PSPL, and the amount of recast diamond. The laser wavelength has a great effect on the modified layer; as shown in Figure 8b, no obvious graphite layer was observed on the surface after ablation with ultraviolet PSPL. Thus, there should be much less graphite generated on the surface after ultraviolet PSPL cutting than infrared PSPL cutting. This is attributed to less thermal impact on CVD-D when it is processed by ultraviolet PSPL, and less diamond material recast on the clearance face from sufficient energy absorption of the ultraviolet laser by vaporized diamond material.

**Figure 9:**
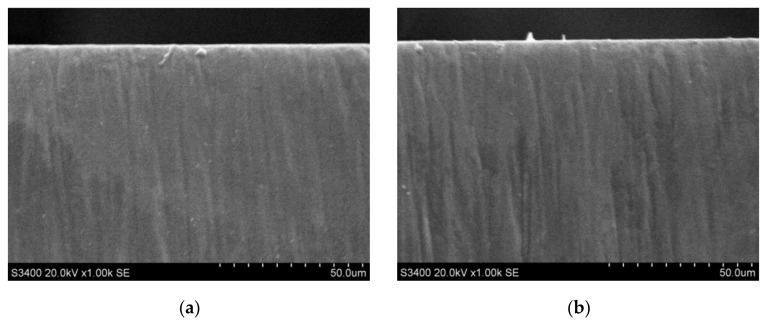
CVD-D micro tools after ultraviolet PSPL cutting with same fluence (12.4 J/cm^2^) and different pulse pitches, (**a**) pulse pitch = 0.5 nm, (**b**) pulse pitch = 5 nm.

The surfaces obtained after ultraviolet PSPL with the same fluence (12.4 J/cm^2^) and different pulse pitches are shown in Figure 9. The surface fabricated with the pulse pitch of 0.5 nm (Ra 149 nm) was much smoother than the surface fabricated with the pulse pitch of 5 nm (Ra 298 nm), and fewer cracks were found on the surface and cutting edge. When the pulse pitch was too large, the PSPL ablated the CVD-D material inadequately and caused deep stripes on the fabricated surface.

Thus, CVD-D micro tools cut with a shorter pulse pitch and ultraviolet PSPL can achieve higher surface quality. Therefore, ultraviolet PSPLs can be used to process diamond semiconductors, optical windows, heat spreaders, and some electronic device elements with good surface quality.

## 4. Case Study

**Figure 10:**
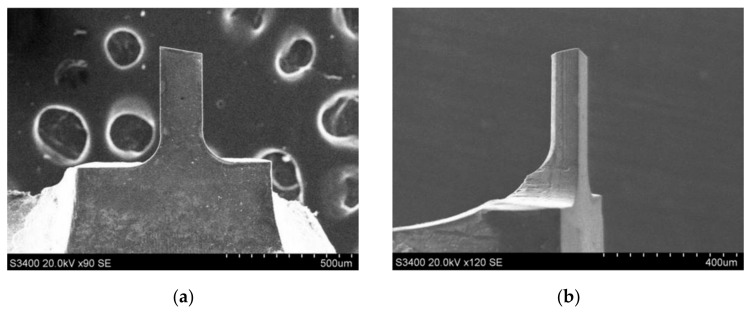

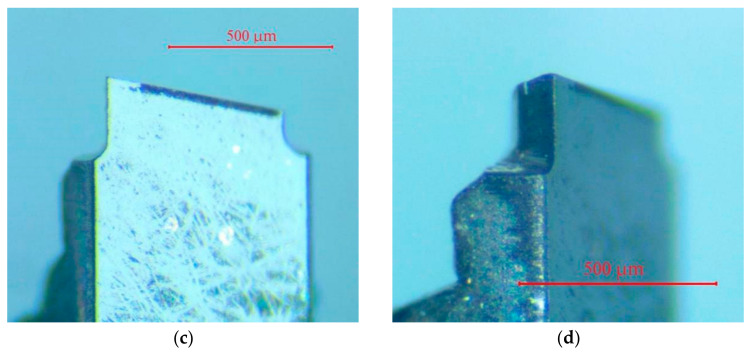
The morphology of (**a**) the CVD-D micro milling tool with the diameter of 200 μm; (**b**) cutting edge and tip of the tool with the diameter of 200 μm; (**c**) the CVD-D micro milling tool with the diameter of 600 μm; (**d**) cutting edge and tip of the tool with the diameter of 600 μm.

Because of the defective result of the ultraviolet laser process on the sharpness of the cutting tool, the quadrilateral CVD-D micro milling tools were manufactured by PSPL with longer wavelengths. Two tools were fabricated with optimized laser parameters: fluence (12.4 J/cm^2^), pulse pitch (2.5 nm), and wavelength (1064 nm), as shown in Figure 10. The side cutting edge and polygonal structure of the tools were fabricated by controlling the A-axis, while the bottom edge is formed by adjusting the B-axis (shown in Figure 2) during laser cutting.

The diameter of the first tool was 200 μm, and its blade length 400 μm; the diameter of the second tool was 600 μm. The SEM and optical microscope (OM) images in Figure 10 indicate a regular cutting edge and good surface quality of the tools. The total PSPL processing of the tool took 10 min, and it was much more efficient than traditional processing methods such as mechanical grinding, which generally take dozens of hours.

The milling experiments were conducted on a three-axis high precision micro milling system developed by NUAA (Nanjing University of Aeronautics and Astronautics). The tool spindle can rotate at 40,000 rpm.

**Figure 11:**
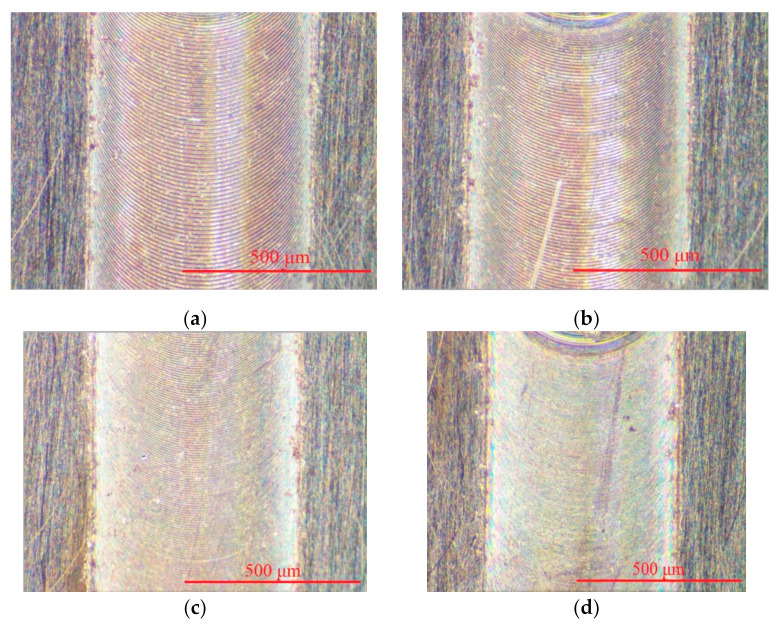

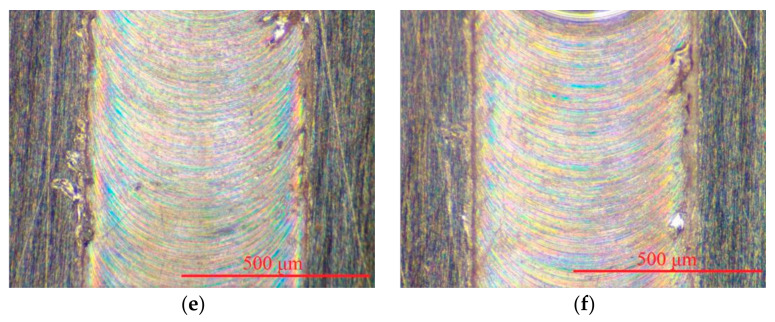
Grooves fabricated by the CVD micro milling tool; (**a**) f_z_ = 12.5 μm; (**b**) f_z_= 10 μm; (**c**) f_z_ = 6 μm; (**d**) f_z_ = 3 μm; (**e**) f_z_ = 2.5 μm; (**f**) f_z_ = 1 μm.

We used oxygen-free copper as the workpiece material, and the micro tool with the diameter of 600 μm had been used for micro milling in this experiment. Grooves of 0.6 mm in width and 0.01 mm in depth were fabricated by the CVD-D micro milling tool at a spindle speed of 20,000 rpm, depth of cut of 5 μm, and different feeds per tooth of 12.5, 10, 6, 3, 2.5, and 1 μm. As shown in Figure 11, the optical microscope images of the grooves indicate a good surface quality and few burrs on the groove edges. The roughness of the micro-milled surface was measured using a Mahr Perthometer M1 roughness measuring instrument. The surface roughness of the grooves were Ra 132, Ra 126, Ra 101, Ra 67, Ra 98, and Ra 122 nm.

The researchers of our lab had fabricated a CVD-D tool with a precision grinding process, and the performance of the cutting tool was studied with regard to micro-milling of oxygen-free copper [22]. The cutting edge radius and nose radius of the CVD-D tool were 2.3 μm and 2.5 μm, respectively. After micro milling of oxygen-free copper, the surface roughness Ra of 53 nm to 140 nm with the different feed per tooth from 1 μm to 5 μm were obtained with the ground CVD-D tool. The results showed that the milling quality of the CVD-D micro tool fabricated with PSPL is very close to the traditional fabricated tool, but the PSPL process is much more efficient than mechanical grinding.

## 5. Conclusions

In this study, the optimization of picosecond pulsed laser cutting of CVD-D micro tools was implemented with the single factor test and variance analysis. The result showed that the PSPL wavelength had the strongest influence on the cutting edge radius of CVD-D. It was also found that the CVD-D micro tool cut with a shorter PSPL pulse pitch could achieve higher surface quality. Finally, CVD-D micro milling tools with the 200 and 600 μm diameters were processed, and micro milling experiments of oxygen-free copper were implemented by the developed CVD-D micro milling tool.

## Figures and Tables

**Table 1:** Parameters of the picosecond pulsed lasers (PSPLs).

Laser system	Infrared PSPL	Ultraviolet PSPL
average output power	100 W	30 W
beam quality (M^2^)	<1.3	<1.3
wavelength	1030 nm	355 nm
pulse duration	<10 ps	<12 ps
min. repetition rate	1 kHz	1 kHz
max. repetition rate	400 kHz	500 kHz
polarization	linearly polarized	linearly polarized
focus diameter	16 μm	16 μm

**Table 2:** Experimental parameters and the corresponding levels.

Laser Cutting Parameters	Fluence (J/cm^2^)	Pulse Pitch (nm)	Wavelength (nm)
Level 1	12.4	0.5	1064
Level 2	19.9	2.5	355
Level 3	24.9	5	

**Table 3:** Analysis of the cutting edge radius.

Number	Fluence (J/cm^2^)	Pulse Pitch (nm)	Wavelength (nm)	R_n_ (μm)
1	12.4	0.5	1064	1.9
2	12.4	2.5	1064	1.4
3	12.4	5	1064	1.5
4	19.9	0.5	1064	2.7
5	19.9	2.5	1064	3
6	19.9	5	1064	3.2
7	24.9	0.5	1064	3.9
8	24.9	2.5	1064	3.5
9	24.9	5	1064	4.1
10	12.4	0.5	355	7.5
11	12.4	2.5	355	6.4
12	12.4	5	355	5.8
13	19.9	0.5	355	9.3
14	19.9	2.5	355	7.8
15	19.9	5	355	7.5
16	24.9	0.5	355	12.2
17	24.9	2.5	355	10.6
18	24.9	5	355	11.1
X_1_	4.1	6.2	2.8	
X_2_	5.6	5.4	8.7	
X_3_	7.5	5.5		
Max-min	3.4	0.8	5.9	

**Table 4:** Variance analysis of cutting edge radius.

Particulars	Flunece (J/cm^2^)	Pulse Pitch (nm)	Wavelength (nm)	Total
Sum of square	5.8	0.38	17.4	23.58
Mean square	2.9	0.19	17.4	20.49
Degree of freedom	2	2	1	5
Contribution (%)	14.15%	0.93%	84.92%	
